# Systematic Analysis of mRNAs and ncRNAs in BMSCs of Senile Osteoporosis Patients

**DOI:** 10.3389/fgene.2021.776984

**Published:** 2021-12-20

**Authors:** Yiyun Geng, Jinfu Chen, Chongfei Chang, Yifen Zhang, Li Duan, Weimin Zhu, Lisha Mou, Jianyi Xiong, Daping Wang

**Affiliations:** ^1^ Shenzhen Key Laboratory of Tissue Engineering, Shenzhen Second People’s Hospital (The First Hospital Affiliated to Shenzhen University), Shenzhen, China; ^2^ School of Biotechnology and Food Engineering, Changshu Institute of Technology, Suzhou, China; ^3^ Guangdong Provincial Research Center for Artificial Intelligence and Digital Orthopedic Technology, Shenzhen, China

**Keywords:** senile osteoporosis, human bone marrow mesenchymal stem cells, whole transcriptome sequencing, non-coding RNA, ceRNA network

## Abstract

Senile osteoporosis (SOP) is a worldwide age-related disease characterized by the loss of bone mass and decrease in bone strength. Bone mesenchymal stem cells (BMSCs) play an important role in the pathology of senile osteoporosis. Abnormal expression and regulation of non-coding RNA (ncRNA) are involved in a variety of human diseases. In the present study, we aimed to identify differentially expressed mRNAs and ncRNAs in senile osteoporosis patient-derived BMSCs via high-throughput transcriptome sequencing in combination with bioinformatics analysis. As a result, 415 mRNAs, 30 lncRNAs, 6 circRNAs and 27 miRNAs were found to be significantly changed in the senile osteoporosis group. Gene Ontology (GO) and Kyoto Encyclopedia of Genes and Genomes (KEGG) analysis were applied to analyze the function of differentially expressed mRNAs and ncRNAs. The circRNA–miRNA–mRNA regulatory network was constructed using the cytoHubba plugin based on the Cytoscape software. Interestingly, circRNA008876-miR-150-5p-mRNA was the sole predicted circRNA-miRNA-mRNA network. The differential expression profile of this ceRNA network was further verified by qRT-PCR. The biological function of this network was validated by overexpression and knockdown experiments. In conclusion, circRNA008876-miR-150-5p-mRNA could be an important ceRNA network involved in senile osteoporosis, which provides potential biomarkers and therapeutic targets for senile osteoporosis.

## Introduction

Senile osteoporosis (SOP) is an age-related skeleton disease characterized by decreased bone mass and strength, which may lead to increased risk of fragility fractures (Raisz, 2005; Brown, 2017). With the increase of longevity, senile osteoporosis has recently become a major chronic metabolic bone disease in the world. Among people over 50 years old, one third of women and one fifth of men are susceptible to suffer from osteoporosis-induced bone fracture (Hendrickx et al., 2015). Bone homeostasis is a dynamic balance mediated by osteoblastic bone formation and osteoclastic bone resorption, and insufficient bone formation could lead to osteoporosis (Kim et al., 2020). Osteoblasts, which are differentiated from bone mesenchymal stem cells (BMSCs), play fundamental roles in bone formation by increasing the amount of matrix and the level of mineralization (Kiernan et al., 2017). In senile, the proliferation and differentiation level of BMSCs significantly reduced, leading to bone formation deficit and osteoporosis (Kiernan et al., 2016). Therefore, it is of great important to investigate the molecular mechanism of impaired BMSCs in aging-induced osteoporosis and search for novel therapeutic targets.

Non-coding RNAs (ncRNAs) are non-protein coding transcripts served as crucial regulators of gene expression and cell fate (Schwarzer et al., 2017), which has been suggested as a novel class of potential diagnostic biomarkers and therapeutic targets (Beermann et al., 2016). The tremendous progress of high-throughput sequencing technologies has resulted in a plethora of studies focusing on ncRNAs, including microRNAs (miRNAs), circular RNAs (circRNAs), as well as long ncRNAs (lncRNAs) (Anastasiadou et al., 2018). As of today, the role of miRNAs in osteogenic differentiation of BMSCs have been widely explored, partially elucidating the pathogenesis of osteoporosis (Ell and Kang, 2014). LncRNAs, defined as linear transcripts of over 200 nucleotides, have also been suggested as therapeutic targets in various human diseases including osteoporosis (Kopp and Mendell, 2018; Chen et al., 2020; Del et al., 2020). Unlike abovementioned linear RNAs, circRNAs are characterized by a stable and closed RNA loops lacking 5′ and 3′ ends. Importantly, the expression of circRNAs is tissue- and cell-specific, indicating that circRNAs could involve in special biological pathways and thus exhibit unique cellular functions (Salzman et al., 2013). Indeed, both lncRNAs and circRNAs are able to act as competing endogenous RNAs (ceRNAs) by targeting miRNAs, and hence regulating the expression of miRNAs’ target genes (Hansen et al., 2013; Tao et al., 2019). Recently, multiple studies have suggested that lncRNAs and circRNAs play significant roles in osteoporosis. For instance, lncRNA GAS5 has been revealed to promote osteogenic differentiation of mouse BMSCs by targeting the miR-135a-5p/FOXO1 axis (Wang et al., 2019). LncRNA Xist functions as a molecular sponge of miR-19a-3p to repress osteogenesis in age-related osteoporosis mouse model (Chen et al., 2020). Downregulation of hsa-circRNA 0006393 has been found in BMSCs from glucocorticoid-induced osteoporosis (GIOP) patients, which was shown to enhance osteogenesis by targeting miR-145-5p/FOXO1 (Wang et al., 2019). CircRNA 0016624 could regulate osteogenic differentiation of BMSCs by targeting miR-98 *in vitro* (Yu and Liu, 2019). However, transcriptome-wide profiling of abnormally expressed ncRNAs in senile osteoporosis as well as their regulatory networks have not been extensively investigated.

To screen the potential ncRNAs involved in senile osteoporosis and explore their molecular mechanism, we systematically analyze the expression profiles of miRNAs, lncRNAs, circRNAs and mRNAs in BMSCs of senile osteoporosis patients and normal individuals by whole transcriptome sequencing. According to the differentially expressed ncRNAs in the SOP group, we found that circRNA008876-miR-150-5p-mRNA represented the only distinctive ceRNA network, which was further validated by molecular biology experiments including qRT-PCR, western blot, overexpression and knockdown. In conclusion, our study established that circRNA008876-miR-150-5p-mRNA could be an important ceRNA network involved in senile osteoporosis, which provides potential biomarkers and therapeutic targets for senile osteoporosis.

## Materials and Methods

### Isolation and Culture of Human BMSCs

Human BMSCs (hBMSCs) of senile osteoporosis were isolated from the bone marrow of discarded bone tissue from male senile osteoporosis patients (79 ± 5.6 years) who underwent joint replacement and diagnosed as SOP with bone minerality tests using Dual Energy X-ray Absorptiometry (Lorente et al., 2012). The normal hBMSCs were obtained using ScienCell (#7500), derived from male normal individuals under 20 years. The hBMSCs were cultured in MesenGro human mesenchymal stem cells medium (StemRD) supplemented with 10% FBS (E510008, Sangon Biotech) at 37°C with humidified 5% CO_2_. The third passage of hBMSCs were utilized for whole transcriptome sequencing. DMSO was ordered from Sangon Biotech (A503039). The entire research plan has been approved by the ethics committee of Shenzhen Second People’s Hospital (The First Hospital Affiliated to Shenzhen University).

### Whole Transcriptome Sequencing

Three SOP patients-derived hBMSCs (BP1, BP2, and BP3) and three normal individuals (BN1, BN2, and BN3) derived hBMSCs were subjected to whole transcriptome sequencing. Total RNA was extracted from hBMSCs using Trizol reagent (Invitrogen) according to the manufacturer’s instructions. After quantification and integrity of RNA samples were verified, rRNAs were removed from total RNA using Ribo-Zero rRNA Removal Kits (Illumina). Then, RNA libraries were constructed with TruSeq Stranded Total RNA Library Prep Kit (Illumina), and the two libraries were then sequenced on HiSeq-4000 (Illumina).

### Construction of ceRNA Network

The miRNA binding regions of ncRNA and mRNA were analyzed using miRanda software (version 0.10.80) and Targetscan software (Release 7.2). The DE miRNAs targeting DE lncRNA, circRNA and mRNA were predicted to form the lncRNA/circRNA-miRNA-mRNA networks.

### GO Enrichment and KEGG Pathway Analysis

Gene function related to senile osteoporosis was analyzed by GO annotation derived from Gene Ontology (www.geneontology.org) in which mRNA were grouped according to biological processes (BPs), cellular components (CCs) and molecular functions (MFs). The related pathways were enriched by KEGG and presented in a scatter plot diagram. We were only interested in biological processes and KEGG pathways showing significance according to the following parameters: *p* < 0.05, FDR <0.05, and enrichment score >1.5.

### Real-Time Quantitative PCR

cDNA was synthesized with PrimeScript™ RT reagent Kit (Takara) using 1ug total RNA after gDNA was erased according to the manufacturer’s instructions. Applied Biosystems 7500 Real-Time PCR Systems was applied to perform amplification reaction using SYBR Premix Ex Taq (Takara). *GAPDH* and *U6* were used as endogenous controls for circRNA008876, osteogenic genes and miR-150-5p expressions, respectively. The RNA expressions were analyzed by 2^-△△CT^ method. The primer sequences were listed in Supplementary Table S1.

### Dual-Luciferase Reporter Assay

HEK293T cells were co-transfected with recombinant plasmids (circRNA BR or circRNA BR Mut) and miR-337-3p mimics or negative control siRNAs (GenePharma). The recombinant plasmids were constructed with psiCHECK™-2 using the primers presented in Supplementary Table S2. Lipofectamine 2000 (Invitrogen) was used for transient transfection according to the manufacturer’s instructions. After 24 h, cells were lysed and Firefly and Renilla luciferase activities were measured by Dual-Luciferase Reporter Assay System (Promega). Biological triplicates were performed.

### ALP Staining

ALP staining was applied to test the osteogenesis of BMSCs after transfection of overexpressing vector or siRNA of circRNA008876 for 7 days according to the ALP staining kit protocol (Beyotime, C3206). Photographs were obtained under microscope (Nikon TE 2000).

### Statistical Analysis

Statistical analyses were performed by the GraphPad Prism 8.0 and SPSS 22.0. Statistical significance of two or more biological replicates were performed by analysis of variance (ANOVA) and *post hoc* test. All results are presented as mean ± SD. *p* < 0.05 was considered statistically significant.

## Result

### Evaluation of hBMSCs Isolated From SOP Model

**FIGURE 1:**
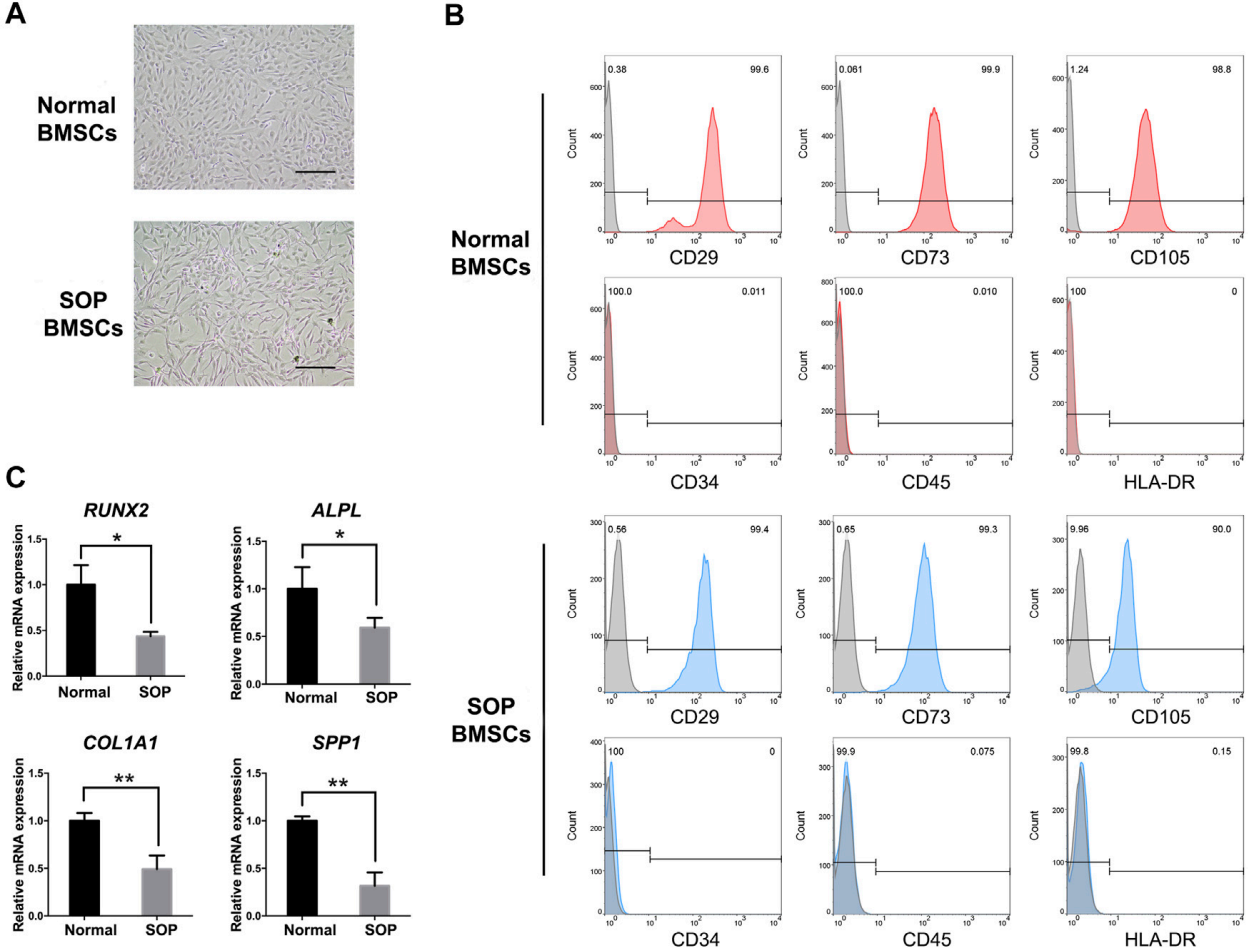
Characterization of hBMSCs from senile osteoporosis patient. **(A)** The morphology of P2 hBMSCs from normal individuals (Normal BMSCs) and SOP patients (SOP BMSCs); **(B)** Stem cell surface markers of hBMSCs were analyzed by flowcytometry. Red (Normal BMSCs) and blue curves (SOP BMSCs) presented positive (CD29, CD73, and CD105) and negative markers (CD34, CD45 and HLA-DR) of hBMSCs, respectively, while grey curves represent isotype controls; **(C)** Expression of osteogenic genes *RUNX2, ALPL, COL1a1* and *SPP1* of hBMSCs from SOP patients and control group were measured by qPCR. *GAPDH* expression was used as an internal control for mRNA expression. Scale bar: 200 μm. Error bars, SEM (n = 3). *p < 0.05, **p < 0.01.

The hBMSCs isolated from SOP patients (passage 0) significantly presented a fibroblastic morphology similar to normal hBMSCs (Figure 1A). The surface markers of mesenchymal stem cells such as CD29, CD73 and CD105 were positively expressed (≥98%) in both groups of hBMSCs, while the hematopoietic markers CD34, CD45 and HLA-DR were negatively expressed (≤0.1%) in both BMSCs as evidenced by flow cytometry results (Figure 1B). Additionally, qPCR results showed that the expression levels of osteogenic genes including *RUNX2*, *ALPL*, *COL1A1* and *SPP1* were significantly reduced in BMSCs from SOP patients as compared to those from normal individuals, indicating the decreased osteogenic differentiation potential of BMSCs in SOP patients (Figure 1C).

### Differentially Expressed lncRNAs, circRNA, miRNAs and mRNAs

**FIGURE 2:**
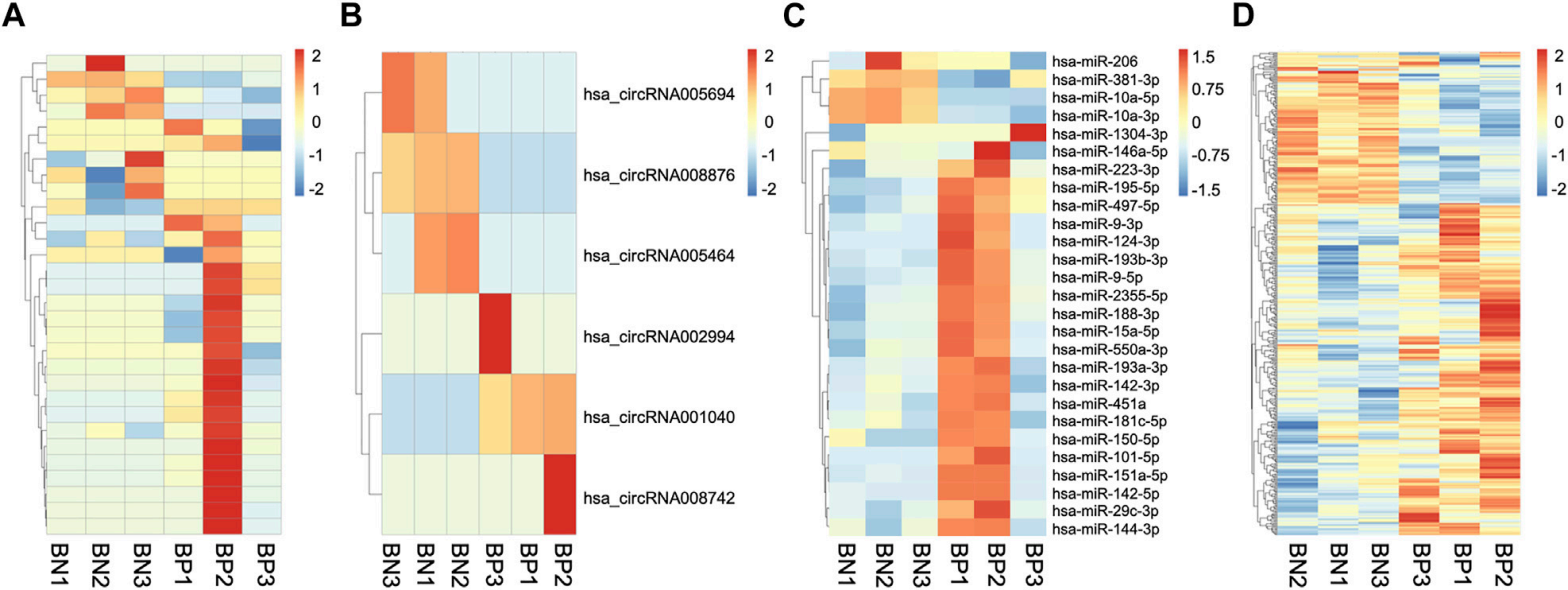
Expression profiles of mRNAs and ncRNAs. Heatmaps clustering of all significantly DE lncRNAs **(A)**, circRNAs **(B)**, miRNAs **(C)** and mRNAs **(D)** in hBMSCs of three senile osteoporosis patients (BP1, BP2, and BP3) and three normal individuals (BN1, BN2, and BN3).

We then compared the expression profiles of ncRNAs and mRNAs of hBMSCs from the two groups. With a fold change cutoff value >2 and *p* value <0.05, 30 lncRNAs (23 upregulated and 7 downregulated), 6 circRNAs (3 upregulated and 3 downregulated), 27 miRNAs (23 upregulated and 4 downregulated) and 415 mRNAs (60 upregulated and 51 downregulated) were identified as differentially expressed (DE) genes between hBMSCs from SOP patients and normal ones. The hierarchical cluster analysis of lncRNA, circRNA, miRNA, and mRNA demonstrated significant differences between hBMSCs from normal (BN) and SOP (BP) groups (Figures 2A–D). Complete information of all DE ncRNAs and mRNAs are provided in Supplementary Information. RNA-seq data were uploaded in Short Read Archive (SRA) of National Center for Biotechnology Information (NCBI) with accession number SRP337202 under Bioproject PRJNA763497.

### Gene Ontology (GO) Enrichment and KEGG Pathway Analysis

**FIGURE 3:**
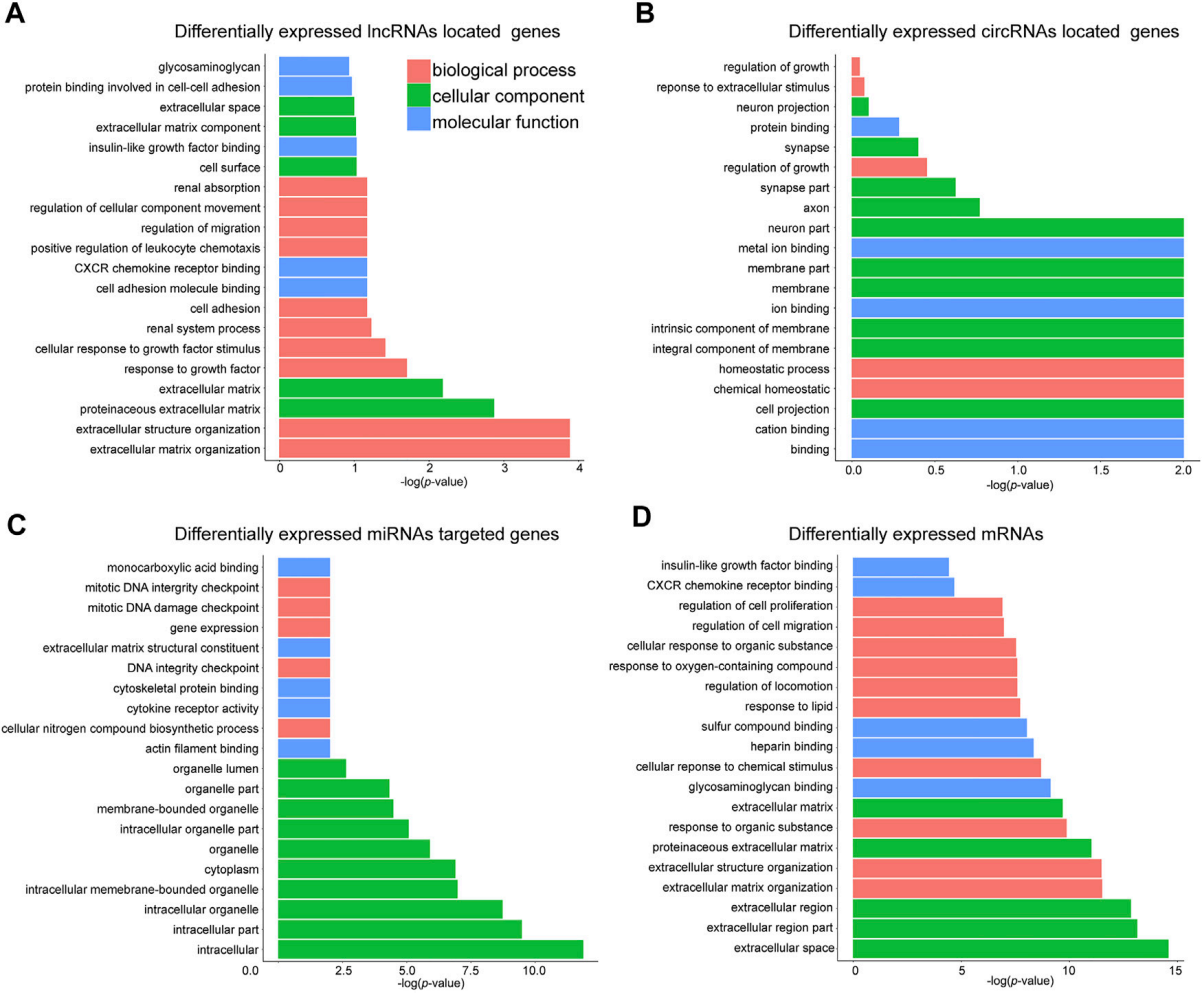
GO analysis of DE mRNAs, lncRNAs, circRNAs and miRNAs. Top 20 terms of Gene Ontology (GO) enrichment analysis regarding **(A)** DE lncRNAs-located genes; **(B)** DE circRNAs-located genes; **(C)** DE miRNAs-targeted genes; and **(D)** DE mRNAs.

We then performed GO enrichment analysis to reveal the key regulators and vital pathways potentially engaged by DE ncRNAs and mRNA in SOP. The top enriched terms regarding biological process (BP), cellular component (CC), and molecular function (MF) of four categories of RNA were shown in Figures 3A–D, respectively. A quick survey on the bioinformatics analysis results revealed that several terms were highly related to osteogenesis. For example, the terms “response to growth factor,” “cell adhesion” and “regulation of migration” are involved in the proliferation of BMSCs, which were remarkably enriched as top biological processes engaged by DE ncRNAs (Figure 3A); similar terms, including “regulation of growth” and “homeostatic process,” were engaged by circRNA-located genes (Figure 3B). For DE miRNA-targeted genes, key processes related to cell division were enriched, including the terms “gene expression,” “DNA integrity checkpoint” and “mitotic DNA damage checkpoint” (Figure 3C)”. For DE mRNAs, three BP terms, namely “regulation of cell migration,” “CXCR chemokine receptor binding,” and “insulin-like growth factor binding,” were related to proliferation and differentiation of BMSCs (Figure 3D).

**FIGURE 4:**
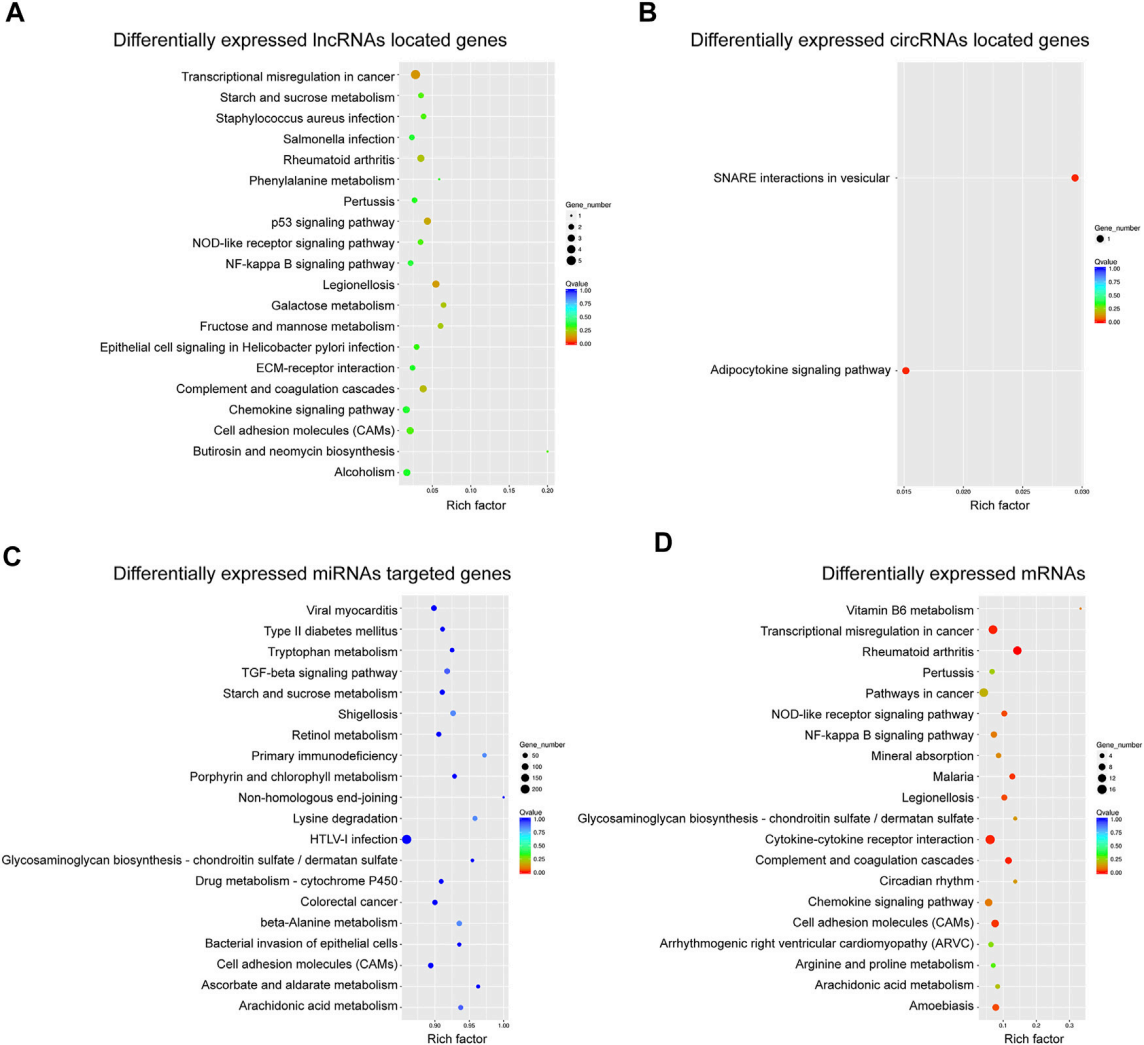
KEGG pathway analysis of mRNAs and ncRNAs. Top 20 KEGG pathways regarding **(A)** DE mRNAs; **(B)** DE lncRNAs-located genes; **(C)** DE lncRNAs-targeted genes; **(D)** DE circRNAs-located genes; and **(E)** DE miRNAs-targeted genes.

We also utilized the Kyoto Encyclopedia of Genes and Genomes (KEGG) database to categorize the DE ncRNAs-targeted genes and mRNAs. The top 20 pathways of KEGG analysis for each category of DE ncRNAs or mRNAs were shown in Figures 4A–D. Among the functional pathways associated with DE miRNAs-targeted genes, we noticed that “TGF-beta signaling pathway” and “cell adhesion molecules (CAMs)” were highly related to osteogenesis (Figure 4C), while “CAMs” were also significantly engaged by DE mRNAs (Figure 4D). In addition, the terms “cytokine-cytokine receptor interaction”, “chemokine signaling pathway” and “mineral absorption” associated with DE mRNAs were also related to osteogenic differentiation of BMSCs (Figure 4D).

### Construction and Analyses of ceRNA Networks

Both lncRNA and circRNA are known to sponge miRNAs to prevent their interactions with target mRNAs, thus exhibiting competitive endogenous RNA (ceRNA) activity. To predict the functions of DE ncRNAs and mRNAs and uncover the potential ceRNA networks regulating the osteogenic differentiation of hBMSCs in SOP patients, lncRNA-miRNA-mRNA and circRNA-miRNA-mRNA networks were constructed by using Targetscan and Miranda softwares.

**FIGURE 5:**
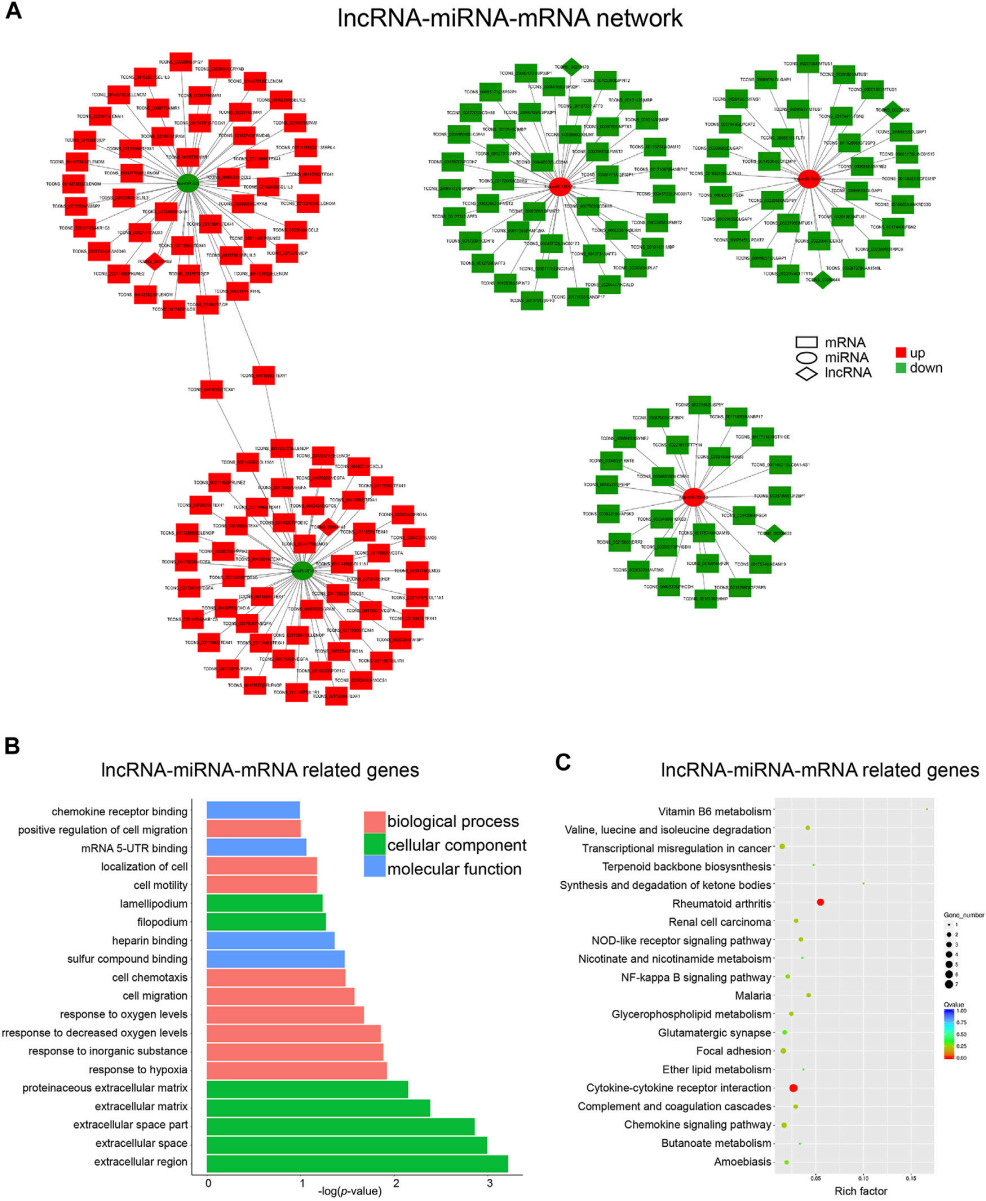
Construction of the lncRNA-miRNA-mRNA Network. **(A)** Network analysis of lncRNA (diamond)-miRNA (round)-mRNA (square). Red and green represent up- and down-regulation, respectively. **(B)** Top 20 GO terms analyzed with mRNAs in lncRNA-miRNA-mRNA network. **(C)** Top 20 KEGG pathway enrichment of mRNAs in lncRNA-miRNA-mRNA network.

As shown in Figure 5A, six lncRNAs were predicted as miRNA sponges, which forms five networks including lncRNA_00214189/miR-206, lncRNA_00056143/miR-381-3p, lncRNA_00211178/miR-2355-5p, lncRNA_00054644/miR-181c-5p, lncRNA_00220556/miR-181c-5p and lncRNA_00206603/miR-150-5p. Among the miRNA-targeted genes in these networks, a number of osteogenesis-related genes were identified including several upregulated genes CCL2, WISP1, WISP2, and one downregulated gene FBN2 (Si et al., 2006; Ono et al., 2011; Smaldone et al., 2011; Córdova et al., 2017). To further understand the biological function of these lncRNA-miRNA-mRNA networks, GO enrichment and KEGG pathway analyses were performed on the target genes. The top 5 cellular component terms were all related to extracellular matrix (Figure 5B), which were highly correlated with the GO enrichment analyses results of DE lncRNAs-located genes and mRNAs (Figure 3). The top biological process terms, such as “response to hypoxia,” “cell migration,” and “cell motility,” are obviously involved in osteogenic differentiation of BMSCs. Moreover, the term “cytokine-cytokine receptor interaction” represents the most remarkable KEGG pathway in these lncRNA-constructed ceRNA networks (Figure 5C), which was also significantly enriched in the KEGG analysis results for DE mRNAs.

**FIGURE 6:**
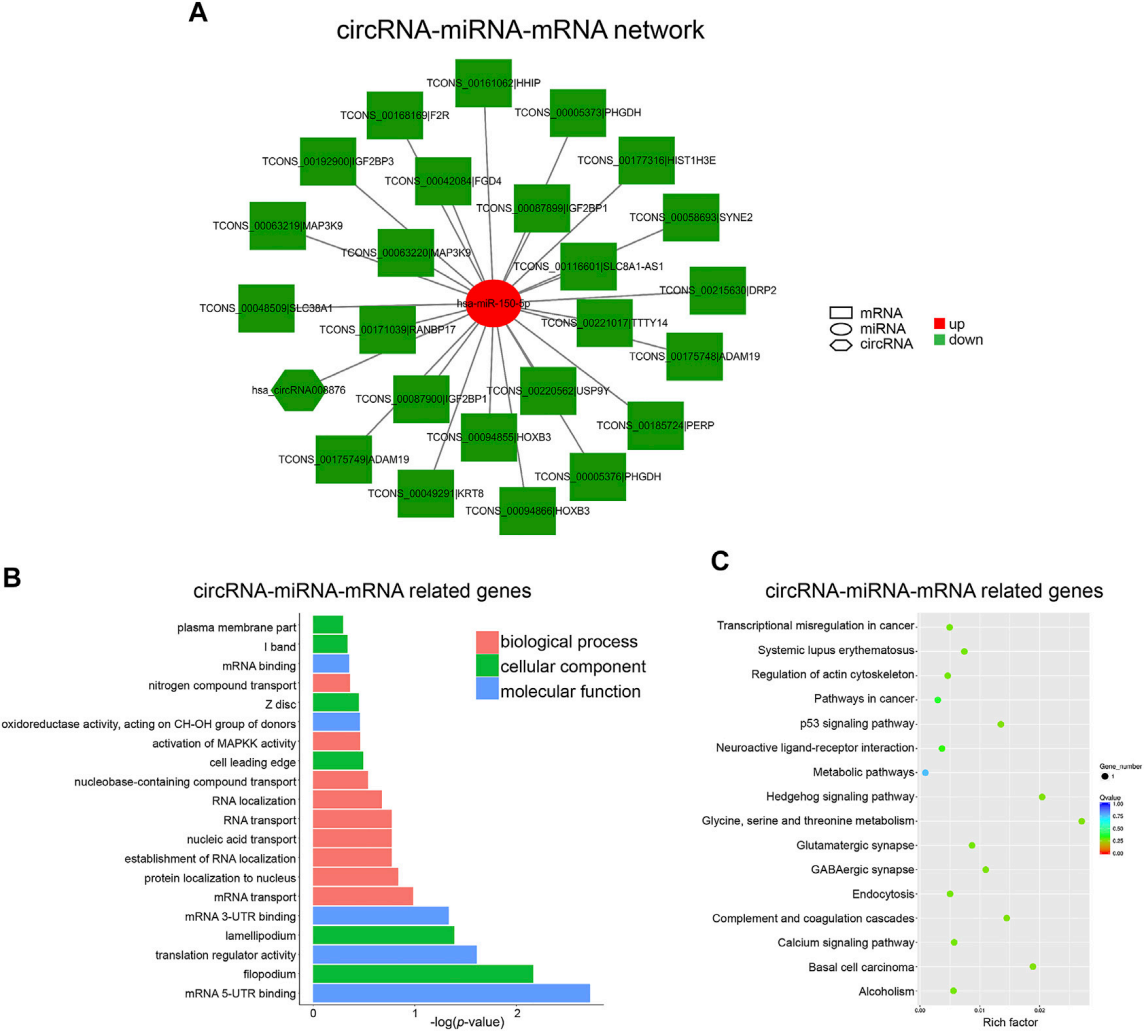
Construction of the circRNA-miRNA-mRNA Network. **(A)** circRNA-miRNA-mRNA network was constructed with down-regulated circRNA_008876 and up-regulated miR-150-5p. **(B)** Top 20 GO terms analyzed with mRNAs in circRNA-miRNA-mRNA network. **(C)** Top 20 KEGG pathway enrichment of mRNAs in circRNA-miRNA-mRNA network.

In the case of circRNA, only one ceRNA network, namely circRNA008876/miR-150-5p, was constructed (Figure 6A). Among the target genes of miR-150-5p, HOXB3 and HHIP have been reported to associate with osteogenesis. GO analysis of target genes of this network showed that “mRNA translation and transport” was the most significant biological function in hBMSCs (Figure 6B). In addition, “p53 signaling pathway” and “Hedgehog signaling pathway” were significantly enriched, which have also been suggested to play important roles in osteogenesis (Tyner et al., 2002; Onodera et al., 2020) (Figure 6C). These results further suggested that both lncRNAs and circRNAs could act as miRNA sponge to regulate the expression of downstream genes, which in turn take part in osteogenic differentiation of BMSCs. Importantly, circRNA008876/miR-150-5p represents a unique circular RNA-based ceRNA network in senile osteoporosis. Therefore, we further explored the biological function and validated the role of circRNA008876 in osteogenic differentiation of hBMSCs.

### Validation of circRNA008876/miR-150-5p ceRNA Network in SOP

**FIGURE 7:**
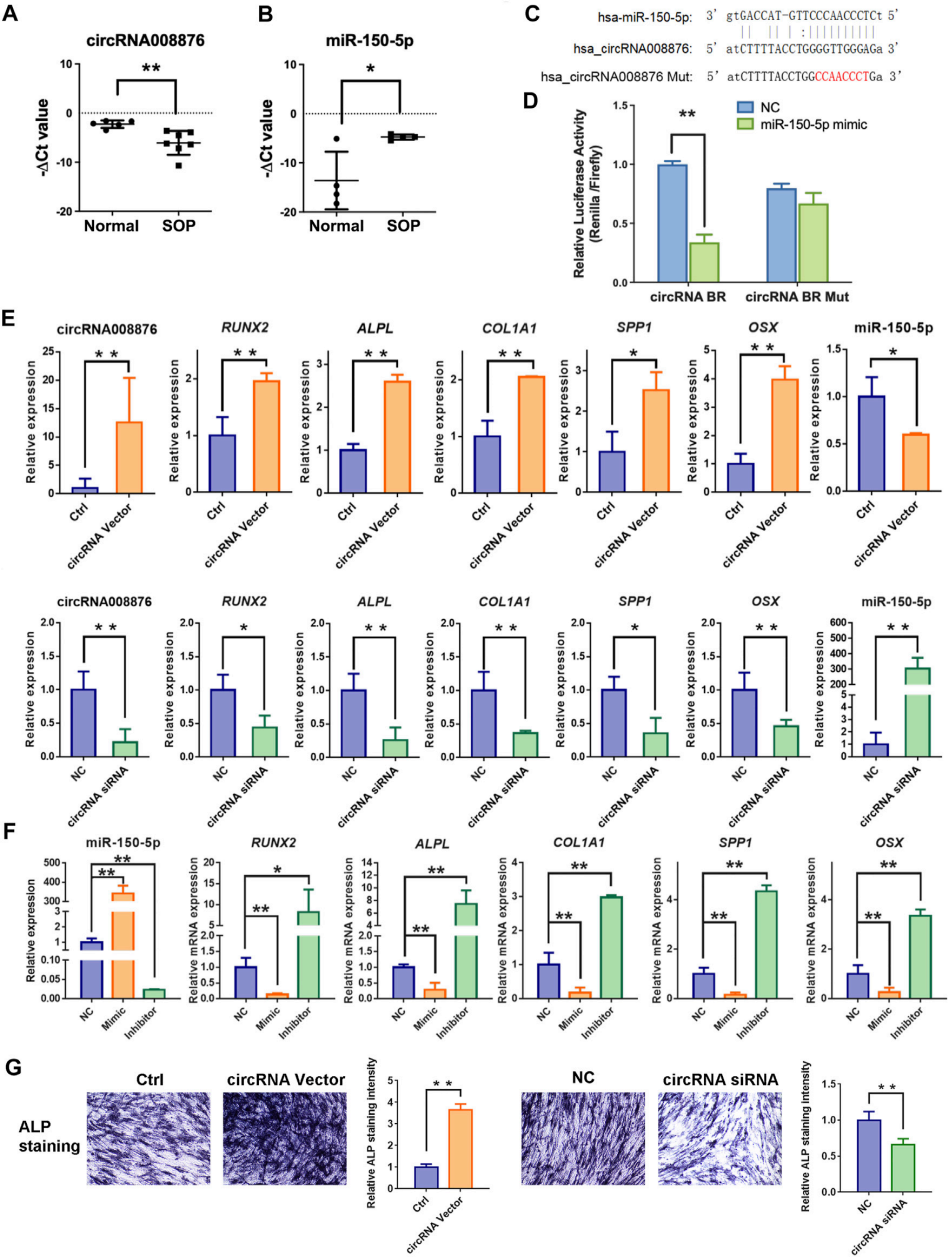
circRNA008876/miR-150-5p regulate osteogenesis of hBMSCs. **(A–B)** Real-time PCR analysis of circRNA008876 **(A)** and miR-150-5p **(B)** expression of hBMSCs from SOP patients or the control group. *GAPDH* and *U6* expression were used as an internal control for circRNA008876 and miR-150-5p, respectively. **(C)** Schematic representation of the wild-type and mutant binding sites of circRNA008876 targeting miR-150-5p. **(D)** HEK293T cells were transfected with psiCHECK™-2 Vector containing a fragment of circRNA008876 binding sites for miR-150-5p, or the corresponding mutant constructs. The effect of miR-150-5p mimics on the corresponding vector luciferase activity was tested. **(E)** hBMSCs were transfected with circRNA008876 overexpressing vector or silencing siRNA. Expression of circRNA008876, miR-150-5p and osteogenesis genes including *RUNX2, ALPL, COL1a1*, *SPP1* and *OSX* were tested by qPCR 3 days after transfection. (F) hBMSCs were transfected with miR-150-5p mimic or inhibitor. Expression of miR-150-5p and osteogenesis genes were tested by qPCR 3 days after transfection. (G) Alkaline phosphatase (ALP) staining on Day 7 of hBMSCs transfected with circRNA008876 overexpressing vector or silencing siRNA. Images were captured under microscope with 100X enlargement. Relative ALP staining intensity were analyzed by ImageJ. Error bars, SEM (n = 3). *p < 0.05, **p < 0.01.

To validate the connection between circRNA00876 and the clinicopathological characteristics of senile osteoporosis, we performed RT-qPCR experiments and the results suggested that reduced expression of circRNA008876 was significantly detected in SOP (Figure 7A), and accompanied with the elevated expression level of miR-150-5p (Figure 7B). To validate the association between circRNA00876 and miR-150-5p, at first, we predicted the possible binding sites (Figure 7C). Then, a dual-luciferase reporter assay was performed by constructing luciferase reporter plasmids harboring the predicted binding sequence of circRNA008876 and its mutation. The luciferase activity of the vector carrying the wild-type binding site of circRNA008876 was significantly inhibited by miR-150-5p mimics, while the luciferase activity did not change in binding site mutated group (Figure 7D), suggesting the direct interaction between circRNA008876 and miR-150-5p. To further confirm the role of circRNA008876/miR-150-5p network in osteogenic differentiation of hBMSCs, we investigated the individual effect of circRNA008876 or miR-150-5p on the expression of several osteogenesis-related genes, including *RUNX2, ALPL, COL1A1, SPP1*, and *OSX* by qPCR. Overexpression of circRNA008876 significantly upregulated the mRNA level of the osteogenic genes, while inhibition of circRNA008876 decreased their mRNA level (Figure 7E). The effect of circRNA008876 on osteogenic differentiation was further confirmed by ALP staining (Figure 7G). Accordingly, up- or down-regulation of miR-150-5p exhibited opposite effect as that of circRNA008876 (Figure 7F), further indicating miR-150-5p as the downstream gene of circRNA008876 in SOP. Taken together, these results demonstrated that circRNA008876 promotes osteogenesis via sponging miR-150-5p, and reduced expression of circRNA008876 leads to impaired osteogenesis in senile osteoporosis through miR-150-5p.

## Discussion

Osteoporosis is a complex bone disease caused by multiple pathogenetic mechanisms, leading to skeletal microarchitectural deterioration and bone mass loss. The pathophysiology and molecular mechanism of senile (type II) osteoporosis (SOP) is different from estrogen deficiency-induced postmenopausal (type I) osteoporosis (PMOP) caused by the activation of osteoclasts. Whereas the osteogenesis deficiency of hBMSCs represents an important pathogenic factor in senile osteoporosis (Rachner et al., 2011), the underlying mechanism remains elusive. Recent studies have shown that ncRNAs including miRNAs, lncRNAs and circRNAs, were significantly involved in osteoporosis development (Jin et al., 2018). Nevertheless, systematic analysis of DE ncRNAs and mRNAs, as well as the ceRNA regulatory networks in hBMSCs from SOP patients, have not be reported. In the present study, hBMSCs from senile osteoporosis patients were isolated, and its decreased osteogenic ability were validated by qPCR. By comparing hBMSCs from SOP patients and normal individuals, 30 lncRNAs, 6 circRNAs, 27 miRNAs, and 415 mRNAs were differentially expressed as suggested by the results of high-throughput RNA-seq.

The function of miRNA in osteogenesis has been extensively investigated in previous studies. Several up-regulated miRNAs of SOP in our study including miR-124-3p, miR-193a, miR-451a, and miR-144-3p have been proven as negative regulators in osteogenesis (Qadir et al., 2015; Lu et al., 2019; Tang et al., 2019; Li et al., 2020; Song et al., 2021). Further studies are needed to investigate the function of other DE miRNAs in SOP. On the other hand, the osteogenic regulatory function of lncRNAs, mainly by targeting and sponging miRNAs, has been reported in recent years.

For example, lncRNA MEG3-miR-133a-3p axis has been suggested to be involved in PMOP through inhibiting osteogenic differentiation of BMSCs (Wang et al., 2017). In addition, lncRNA H19 was found to be a positive regulator of osteogenesis in PMOP via miR-532-3p/SIRT1 axis (Li et al., 2021). In age-related osteoporosis, lncRNA Xist has been reported to inhibit osteogenesis by targeting and sponging miR-19a-3p (Chen et al., 2020). Nevertheless, global investigation and functional studies of lncRNAs in senile osteoporosis is still lacking. In this study, we surprisingly found that the expression of lncRNA_00218,705|XIST also increased in SOP patients’ BMSCs. On the other hand, we revealed several additional significantly up-regulated lncRNAs including lncRNA_00218,705|XIST, lncRNA_00090434|AC144831.1, and lncRNA_00214,189|GS1-358P8.4, as well as several down-regulated lncRNAs such as lncRNA_00211,178|LINC01410 and lncRNA_00219,655|MIR503HG in SOP patients. These lncRNAs could be potential clinical biomarkers which need further validation. Recently, the role of circRNAs in osteoporosis and osteogenesis have also been investigated. For example, has_circ_0001275 has been proven to be a potential diagnostic biomarker for PMOP (Zhao et al., 2018), and some DE circRNAs have been detected in various biological systems, including rBMSCs (Li et al., 2017), human periodontal ligament stem cells (Zheng et al., 2017), and MC3T3-E1 cells (Qian et al., 2017). Unfortunately, the underlying mechanism has yet not been fully explored. Moreover, global profiling of DE circRNAs in senile osteoporosis has not been reported yet. Here, we successfully identified 6 novel DE circRNAs that may take part in senile osteoporosis, all of which have not been studied in osteoporosis before.

The functions of DE ncRNAs and mRNAs in senile osteoporosis pathology was pedicted by GO enrichment (BP, CC, and MF) and KEGG pathways analyses. As we known, extracellular matrix mineralization is an essential progress in bone formation (Murshed, 2018). According to the results of GO enrichment analysis, we noticed that extracellular matrix is one the most significant terms of CC analysis regarding DE mRNAs, DE lncRNAs-located mRNAs, and DE miRNAs-targeted mRNAs. Meanwhile, CXCR chemokine receptor binding were significantly enriched in MF analysis regarding both DE mRNAs and DE lncRNAs-located mRNAs, validating previous results regarding the role of CXCR-4 in recruiting MSCs in bone repair (Zhang et al., 2017). Moreover, KEGG pathway analysis revealed that the adipocytokine signaling pathway was significantly engaged by DE circRNAs-located mRNAs, which implies that DE circRNAs may participate in the balance of osteogenesis and adipogenesis of hBMSCs. It is well-established that the shift from osteogenesis to adipogenesis is a major pathological progress of SOP (Chen et al., 2016). Noticeably, lncRNAs may regulate p53 signaling to affect hBMSCs differentiation, as p53 has been proved to promote osteogenesis in mesenchymal stem cells (Tataria et al., 2006).

In general, the regulatory function of cell differentiation by ncRNAs are mainly through the ceRNA network. In this study, five lncRNA-miRNA-mRNA networks were constructed, among which miR-206, miR-381-3p were down-regulated, while miR-2355-5p, miR-181c-5p, and miR-150-5p were up-regulated. Previous reports suggested that miR-206 and miR-381-3p were able to inhibit osteogenic differentiation of BMSCs (Chen et al., 2019; Long et al., 2019), which were in conflict with our results. The effect of miR-2335-5p, miR-101c-5p in osteogenic differentiation has never been reported. In addition, miR-150, sponged by lncRNA TCONS_00206,603, was proved to serve as a negative regulator for osteoblasts (Moussa et al., 2021), which was found to be upregulated in SOP patients in our study. However, we have not detected any downregulation of lncRNA TCONS_00206,603 in SOP patient. Interestingly, down-regulation of circRNA008876 was predicted to be a unique circRNA sponge for miR-150-5p. Thus, we systematically validated the role of circRNA008876 in BMSCs osteogenesis by targeting and sponging miR-150-5p. However, there are some limitations in this study. That is, the molecules downstream of the circRNA008876/miR-150-5p axis are unknown, and *in vivo* pro-osteogenesis effect induced by circRNA008876 overexpression is unclear. To be noted, the abovementioned genes identified in this study, mainly including lncRNAs and circRNAs, did not overlap in previous studies. These results can be attributed to the differences in patients and ethnicity, which need further investigation.

## Conclusion

In the present study, we systematically explored the role of differently expressed ncRNAs and mRNAs in hBMSCs isolated from senile osteoporosis patients by whole transcriptome sequencing. The ceRNA regulatory networks of lncRNA/circRNA-miRNA-mRNA were constructed to further investigate the function of ncRNAs in senile osteoporosis. Among them, circRNA008876 was identified as a unique and critical circRNA in the occurrence and development of senile osteoporosis and might serve as a potential diagnostic biomarker for senile osteoporosis. Overall, our findings provide an insight into the molecular mechanisms of senile osteoporosis in the view of ncRNAs, which may offer novel therapeutic targets for clinic treatment of senile osteoporosis.

## Data Availability

The RNA sequencing datasets can be found in SRA of NCBI with accession number SRP337202 under BioProject PRJNA763497. The data are accessible with the following link: https://www.ncbi.nlm.nih.gov/sra/PRJNA763497.
