# Supplementary material for: Systematic Analysis of mRNAs and ncRNAs in BMSCs of Senile Osteoporosis Patients
Source: Front Genet. 2021 Dec 20;12:776984. doi: 10.3389/fgene.2021.776984 (PMC8721150; doi:10.3389/fgene.2021.776984)
Supplement: Supplementary file 1 [file DataSheet1.docx]

1. **Supporting Information Tables**

**Table S1 Primer sequences used for real-time quantitative PCR analysis**

| Gene | Strand | Primer sequences |
| --- | --- | --- |
|  |  |  |
| *RUNX2* | F | TACCTGAGCCAGATGACG |
|  | R | CAGTGAGGGATGAAATGC |
| *ALPL* | F | CAACCCTGGGGAGGAGAC |
|  | R | CACTAGCAAGAAGAAGCCTTTGG |
| *COL1A1* | F | GCCGTGACCTCAAGATGTG |
|  | R | GCCGAACCAGACATGCCTC |
| *SPP1* | F | ATTCTGGGAGGGCTTGGTT |
|  | R | AGTCTGGTCCCGACGATG |
| *OSX* | F | CCTCTGCGGGACTCAACAAC |
|  | R | AGCCCATTAGTGCTTGTAAAGG |
| *GAPDH* | F | GGAGCGAGATCCCTCCAAAAT |
|  | R | GGCTGTTGTCATACTTCTCATGG |
| *circRNA008876* | F | GTGGAATCAAGGGCTAGGAATG |
|  | R | AAGTTTGAAGCTAGGCGCAGT |

**Table S2 Primers for recombinant plasmids for dual-luciferase Reporter Assay**

| Gene | Strand | Primer sequences |
| --- | --- | --- |
|  |  |  |
| *circRNA-BR* | F | CCGCTCGAGCACAGTCTCTCTGGGATTATCT |
|  | R | ATAAGAATGCGGCCCATTCCTAGCCCTTGATTCC |
| *circRNA -BR MT* | F | TTACCTGGCCAACCCTGAAGAGAAGAG |
|  | R | CACTTCTTCAGGGTTGGCCAGGTAA |

**2. Procedures for Biological Experiments**

**2.1 Flowcytometry**

Passage 2 of hBMSCs were applied for mesenchymal stem cell surface marker identification by flowcytometry. hBMSCs were incubated with primary antibodies CD34, CD105, CD29, CD73, CD45 and HLA-DR and then incubated with phycoerythrin (PE) conjugated secondary antibody following the manufacturer’s instructions (HUXMX-09011, Cyagen, China). Negative and isotype controls were performed. Immunofluorescence of cells was measured by flowcytometry (Beckman Coulter) after incubation.

**2.2 miRNA qPCR**

Total RNA of hBMSCs was extracted using Trizol reagent (Invitrogen) according to the manufacturer’s protocol. Reverse transcription reaction was carried using Bulge-Loop^TM^ miRNA qRT-PCR kit (Ribo Bio, China) for miR-150-5p and *U6.* qPCR was performed using SYBR Premix Ex Taq (Takara) by Applied Biosystems 7500 Real-Time PCR Systems. *U6* was used as the internal control for miR-150-5p. The RNA expressions were analyzed using 2^−ΔΔCt^ comparison method. The primers of miR-150-5p and U6 for qPCR were included in Bulge-Loop^TM^ miRNA qRT-PCR kit (Ribo Bio, China).
